# Case of Compound Fracture of the Thigh, with Other Severe Wounds—Tetanus, with Death on the Eleventh Day

**Published:** 1839-04-20

**Authors:** 


*Case of Compound Fracture of the Thigh, with other Severe Wounds—Tetanus, with Death on the eleventh day.*

A. H., a shoemaker, aged sixteen years, was admitted into the hospital April 7th, 1839, having been injured by a rail-road car a few hours previously, the wheel passing over his right leg and grazing the left knee. The right thigh was fractured in its lower third, with a lacerated wound an inch in extent, situated on the outer part of the thigh, opposite to the fracture. There was a lacerated wound of the right leg, commencing on the inner side of the tibia, a little below its tubercle, and extending eight inches downward. This, though deep, did not injure either the bone or any of the large vessels. A wound of about three inches in extent, tearing up the integuments to within an inch of the patella, existed on the inner part of the left knee. When admitted, the boy was very pale, with a weak fluttering pulse, and cold extremities. Brandy punch, with an opiate, was given, and sinapisms applied to the abdomen. There being very little hæmorrhage, adhesive strips with lint, were applied and after the ends of the bone had been placed in apposition, by means of extension and counter-extension, a light bandage, from the toes upwards, and the leg placed in a long fracture-box. The left knee was dressed with strips and lint, and placed upon a pillow.

8*th.*—After a consultation, it was determined that, from the prostration, it was inexpedient to amputate. Delirious during night of 7th. To-day his pulse is 145, and weak. The skin is warm; tongue moist, and covered with a white fur; does not complain of pain; passes his water freely; dressings untouched. ℞. Mist. Neutr. ℥ss., every two hours; Tr. Opii gtts. xv., every four hours. To have a small quantity of brandy punch through the day. Left knee poulticed.

10*th.*—Has slept pretty well during the last two nights. Pulse stronger; skin natural; to continue same remedies. The first bandage was removed from the right leg, and, without disturbing the lint on the wounds, that of Scultetus applied.

11*th.*—Pulse 121, and stronger; skin warm; tongue covered with white fur; passed a good night; delirium entirely gone; appetite very good; having had no stool, he took some salts and magnesia, which operated freely; dressings of right leg undisturbed, as he has no pain; left knee poulticed twice a day; to have quinine gr. j., every two hours; chicken and porter.

12*th.*—Same as at last note. The bandage being removed from right leg, the pledget over the wound in the thigh was found firmly adherent; and as there was no inflammation, was left untouched. The edges of the wound in the leg are sloughing, with a free discharge of pus from the whole surface. Cerate was applied, and over that the Scultetus bandage.

13*th.*—Passed a good night, without pain. Pulse 128, and of moderate strength; skin cool and soft; tongue moist, and but slightly furred. The lint being removed from the wound of the thigh, this was found to look very well; no inflammation or collection of pus; an adhesive strip and lint were again applied. The wound in the leg as before. The wound of the left knee has been sloughing somewhat; this is, however, now arrested, and it is granulating. The joint is not opened. Continue laudanum, porter, quinine, &c.

15*th.*—Doing well. Pulse 128, and of moderate force. Skin natural; tongue slightly furred; bowels open by magnesia; appetite good; wound in thigh as before; that of the leg looks very well; pus healthy.

On the evening of the 16th he was attacked with trismus. The head is directed backward, the mouth closed, and he can with difficulty swallow. There is pain in the back of the neck; intelligence good; senses perfect; pupils natural. He can still, with some effort, flex the head and protrude the tongue, though it is very slowly, and with considerable difficulty. Back of neck to be rubbed with turpentine, and a blister, 4 by 6 inches, to be applied. Through the night he took 395 gtts. of laudanum, which had the effect of contracting the pupil, and relieving somewhat the pain in the neck.

17*th.*—6 A. M. Has as yet had no general spasm. Pulse 140, and weak; skin warm; tongue covered with yellow fur; pupils somewhat contracted; trismus as before; can still protrude the tongue. Up to 9 A. M. took 275 gtts. of laudanum. At this time, while dressing the right leg, it was seized with violent spasms, upon which ʒj. of laudanum was given, which, for the time, arrested them. The wound in the right leg is now discharging a thinner pus than yesterday, though in other respects it has much the same appearance. An enema was given, which operated freely.

10½ A. M. Trismus more violent; senses perfect; still complains of the pain in the neck; the pulse being very weak, and the skin cool, brandy ʒj. in sago, was given every half hour; to have an enema of gtts. c. of laudanum; to have a teaspoonful of laudanum, whenever the general spasm becomes violent.

6½ P. M. Has taken ʒvss. of laudanum since last note. Trismus still continues, though able to protrude the tongue, which is moist; skin warm and moist; complains of but little pain; pupils contracted; senses natural; no stupor or inclination to sleep; has at intervals of about fifteen minutes a slight general spasm. Pulse 150, and weak, irregular.

He was after this period attacked with some severe general spasms, for which he took ʒvss. of laudanum up to 12 o'clock the same night, at which period he died.

